# The dynamics of piRNA expression in *Blattella germanica* ovaries

**DOI:** 10.1371/journal.pone.0347365

**Published:** 2026-10-06

**Authors:** David Pujal, Guillem Ylla, Agustina Pascual, Josep Bau, Maria-Dolors Piulachs

**Affiliations:** 1 Institute of Evolutionary Biology (CSIC- Universitat Pompeu Fabra), Barcelona. Spain; 2 Department of Biosciences, University of Vic - Central University of Catalonia, Vic, Barcelona, Spain; 3 Faculty of Biochemistry, Biophysics and Biotechnology, Jagiellonian University, Kraków, Poland; 4 Centro de BioInvestigaciones (CeBio-CICBA), Universidad Nacional del Noroeste de la Provincia de Buenos Aires (UNNOBA), Pergamino, Argentina; 5 Centro de Investigaciones y Transferencias del Noroeste de la Provincia de Buenos Aires (CITNOBA-CONICET), Pergamino, Argentina; University of Bari: Universita degli Studi di Bari Aldo Moro, ITALY

## Abstract

The cockroach *Blattella germanica* possesses panoistic ovaries, in which oocytes lack nurse cells and therefore need to rely on their own transcriptional activity to support embryogenesis. Ovarian development in this species involves the development of a single basal ovarian follicle (BOF) per gonadotropic cycle, a process strictly regulated by endocrine signals, primarily juvenile hormone and ecdysone, which act at both the transcriptional and translational levels. In addition, transcriptional activity in these ovaries is necessary for both regulating and genome protection, and at this level, PIWI-interacting RNAs (piRNAs) play an essential role. Although insect ovaries are known to be particularly rich in piRNAs, their function in ovary maturation is still not well defined. For this purpose, we characterize the piRNA expression dynamics in the *B. germanica* ovary, which shows coordinated fluctuations. The piRNA expression remains stable in previtellogenic ovaries, whereas vitellogenic ovaries show pronounced changes. Moreover, vitellogenic ovaries exhibit reduced piRNA diversity due to strong enrichment of a subset of highly expressed piRNAs. Our data show that although piRNAs predominantly map to transposable elements, particularly LINEs, there is a notable increase in mRNA-derived piRNAs toward the end of the cycle. Our results indicate that piRNAs may play regulatory roles in modulating both TEs and mRNAs during BOF maturation, likely linked to changes in the follicular cell program.

## Introduction

Ovarian development in insects is a complex and highly coordinated process regulated by molecular signaling cascades and hormonal control [1–3]. Using *Blattella germanica* as a model, we investigate a new regulatory layer in insect oogenesis mediated by Piwi-interacting RNAs (piRNAs).

piRNAs are small non-coding RNAs (sncRNAs) initially identified in the male germline of *Drosophila melanogaster* [4,5]. Since then, they have been described in many eukaryotes [6]. Their canonical function involves the regulation of transposable elements (TEs), from which they can also originate [5–8], thereby acting as essential genome guardians [9]. However, accumulating evidence has revealed that the piRNA pathway extends beyond TE silencing. In insects, piRNAs have been involved in growth regulation, gonadal and wing development, embryo patterning, and sex determination [9–13]. These findings show the importance of piRNAs as regulatory molecules, with some cases showing functional parallels to microRNAs [6].

In a previous study based on *B. germanica* development, we identified a total of 866,980 unique piRNAs across 11 developmental stages, from egg and embryo through successive nymphal instars to adult females [14]. The abundance of piRNAs detected in unfertilized eggs suggests a significant role for piRNAs in ovarian physiology. These piRNAs appear to accumulate during oocyte growth, supporting ovarian maturation and potentially influencing early embryogenesis. Notably, *B. germanica* oogenesis is characterized by the growth of a single basal ovarian follicle (BOF) per ovariole in each gonadotropic cycle. Thus, most molecular changes observed in the ovary correspond to BOF development (reviewed in 2 and 3), a process tightly regulated by juvenile hormone (JH) and ecdysone (Fig 1).

**Fig 1:**
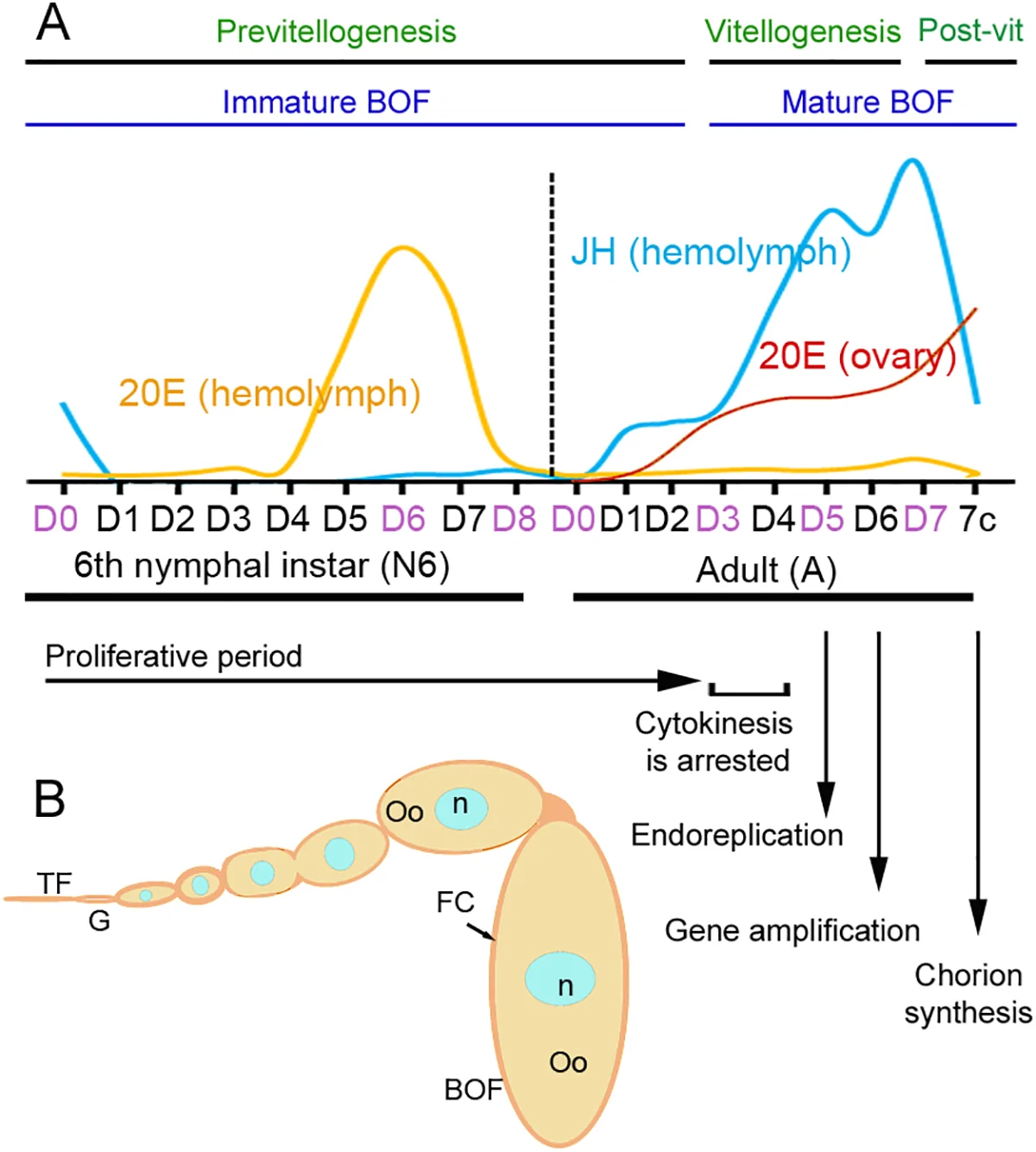
The oogenesis in *Blattella germanica.* **A.** Profiles of juvenile hormone (JH, blue) and 20-hydroxyecdysone (20Ec, yellow) in the hemolymph of last instar nymphs (N6) and adult females (A; 7c indicates females with ovaries in choriogenesis) (see [1–3]). The profile of 20E content in the ovaries is represented in red [1]. The letter D, followed by a number, indicates the age of the individual in days within the instar. The days chosen for library construction, which coincide with significant changes in both hormones, are highlighted in violet. Ovaries from newly emerged last instar nymphs (N6D0) with JH and no 20E, N6D6 with the highest levels of 20E in the hemolymph and the absence of JH, and N6D8 with low levels of both hormones, before the molt to the adult, were chosen. The adult ovaries included were: just after the molt (AD0), when hormone levels were very low. AD3, when JH levels in the hemolymph increase and induce vitellogenesis. AD5, in the vitellogenic period when JH in the hemolymph is at high levels, and the content of 20E in the ovary is increasing. AD7, just at the end of the cycle before chorion formation. JH levels reach their maximum, declining significantly during these 24 hours, while 20E content is the highest in the ovary. The progression of the follicular cell cycle in the BOFs is indicated below the plot, from the first day of the sixth instar nymph (N6D0) to chorion formation in the adult (AD7c). **B**. Schema corresponding to a *B. germanica* ovariole. BOF: basal ovarian follicle; FC: Follicular cells; G: germarium; n: oocyte nucleus; oo: oocyte; TF: terminal filament.

To elucidate piRNA function during oogenesis, we used small RNA-seq to identify the piRNAs expressed in *B. germanica* ovaries throughout the gonadotropic cycle by sampling key developmental stages. These included ovaries with previtellogenic (immature), vitellogenic, and post-vitellogenic (mature) BOFs (Fig 1A). We selected seven stages defined by hormonal peaks (JH or ecdysone) and by characteristic morphological changes in the follicular epithelium in the BOF (Fig 1A, B).

Three stages corresponded to the last (6th) nymphal instar, when prothoracic-gland-derived ecdysone predominates in the hemolymph [15]. During this instar, the basal oocyte becomes fully transcriptionally active and initiates the synthesis and organization of macromolecules required later for embryogenesis. The selected nymphal stages were: newly emerged last-instar nymphs, marking the onset of the first gonadotropic cycle [16]; 6-day-old last-instar nymphs, when the number of ovarian follicles per ovariole is established, the germarium cell differentiation is arrested [17], and a hemolymph peak of 20-hydroxyecdysone (20E) occurs, activating the ecdysone signaling pathway [15,17,18]; 8-day-old last-instar nymphs, just before the imaginal molt, when the basal oocyte has completed the accumulation of mRNAs and proteins required for embryogenesis [19] (Fig 1A).

After adult emergence, ovarian physiology is governed primarily by JH, which triggers the onset of vitellogenesis and BOF growth [2,20] (Fig 1B). In adults, ovarian piRNAs were analyzed at four stages: newly emerged adults, before the rise of JH in the hemolymph; 3-day-old adults, when the cytokinesis in BOF follicular cells arrests, ovarian ecdysone synthesis begins, and an increase in JH induces vitellogenesis in the fat-body; 5-day-old adults, when follicular cells become binucleated and polyploid [16,21], JH levels peak [22], patency is established, and ovarian 20E increases to activate early chorion gene expression [17]; 7-day-old adults, a post-vitellogenic stage in which choriogenesis is completed [21] just before ovulation and oviposition; At this moment JH levels decline [22], while ovarian 20E reaches its maximum [15] (Fig 1A).

Our results reveal a substantial proportion of ovarian piRNAs originating from genic regions. Their abundance varies during BOF maturation, increasing markedly in pre-oviposition stages while remaining relatively stable in immature ovaries. These findings suggest that piRNAs could play regulatory roles in modulating both TEs and mRNAs during BOF maturation, likely related to changes in the follicular cell program.

## Materials and methods

### Cockroach colony and sampling

Adult and last nymphal instar females of the cockroach *B. germanica* (L.) were obtained from a colony fed ad libitum on Panlab 125 dog chow and water, reared in the dark at 29 ± 1 °C and 60–70% relative humidity. Females were sampled at seven developmental stages: 1) newly emerged last instar nymphs (N6D0); 2) 6-day-old last instar nymphs (N6D6); 3) 8-day-old last instar nymphs (N6D8); 4) newly emerged adults (AD0); 5) 3-day-old adults (AD3); 6) 5-day-old adults (AD5); 7) 7-day-old adult females in choriogenesis (AD7). All dissections were conducted on specimens anesthetized with carbon dioxide for one minute to prevent any secondary effects of the anesthesia. Tissues were stored at −80 ºC until use.

### Small RNA extraction

Total RNA was isolated using the GenElute Mammalian Total RNA Kit (Sigma, Madrid, Spain). RNA quantity and quality were estimated by spectrophotometric absorption at 260/280 nm in a Nanodrop Spectrophotometer ND-1000® (NanoDrop Technologies, Wilmington, DE, USA).

### qRT-PCR expression studies

For expression studies, 400 ng of each RNA extraction was reverse transcribed using the 1st Strand cDNA synthesis Kit (Agilent Technologies) to obtain cDNA. For qRT-PCR, the forward primer was designed using the full piRNA sequence, while the universal primer from the Agilent 1st Strand cDNA synthesis Kit was used as the reverse. The *U6* small nuclear RNA was used as a reference for expression studies [23]. qRT-PCR was performed as explained before [10]. Expression levels of small RNAs were calculated relative to the reference gene, using the 2-ΔCt method [21]; [24]. Results are given as copies of small RNA per 1000 copies of *U6.* The primer sequences are detailed in S1 Table.

### Small RNA libraries preparation

Each library was constructed using the two ovaries from a single individual. The concentration and purity of the small RNA were assessed spectrophotometrically with a NanoDrop. Following this, the quality of the small RNA intended for library preparation was evaluated using an Agilent 2100 Bioanalyzer, employing the Small RNA assay and the 2100 Expert software from Agilent Technologies. Libraries were prepared and sequenced by BMK (Münster, Germany) using the Illumina Novaseq 6000 (PE50, n = 3), except for AD3 samples (SE50, n = 4). Small RNA-seq data are publicly available at the NCBI Gene Expression Omnibus [25] under the accession code GEO: GSE255380. As a reference genome for the analyses, the *B. germanica* assembly (GCA_003018175.1) generated by Harrison and colleagues [26] was used.

### Annotation of *Blattella germanica* transposable elements

*B. germanica* transposable elements (TEs) were identified using the EarlGrey TE annotation pipeline (v5.1.1; Docker image tobybaril/earlgrey_dfam3.7:latest). The Arthropoda repeat library from Dfam (v3.8) was used to mask known repeats, and TE sequences shorter than 100 bp were removed to generate the final repeat annotation. Gene models were adjusted to remove all overlaps with TE features using bedtools (v2.31.1) [27], and the resulting non-overlapping gene and TE annotations were used for downstream analyses.

### piRNA identification

Library quality was assessed using FastQC (v0.12.1) [28]. Sequencing adapters were removed from the samples using Trimmomatic (v0.39, parameters: ILLUMINACLIP: TruSeq2-PE.fa: 2:30:10 LEADING:20 MINLEN:18) [29], and the trimmed libraries were subsequently subjected to another round of quality control with FastQC. Potential piRNA reads were then selected using Cutadapt (v3.5) [30], with a read length threshold set between 26 and 31 nucleotides. Reads that aligned to *B. germanica* miRNAs and other types of small RNAs (rRNA, tRNA, snRNA) were excluded from the analysis. The remaining reads were aligned to the *B. germanica* genome assembly (GCA_003018175.1) [26] with Bowtie2 (v2.4.4, parameters: -k 1, --score-min L,0,0) [31]. Only the reads that mapped with no mismatches were retained, while mapped reads containing one or more mismatches were removed using samtools (v1.19.2) [32] by selecting alignments with “NM = 0”.

To obtain a more consistent set of *B. germanica* piRNAs, reads mapping to the same genomic locus and sharing the first 26 nucleotides at the 5’ end, differing only in their length at the 3’ end, were collapsed, and the sequence with the highest expression was taken as the representative. The expression of the representative and each 3’ variant were combined to determine the total expression of the piRNA. Then, extremely infrequent piRNAs with overall expression below 10 reads across all libraries were filtered out. The piRNAs obtained after this collapsing and filtering step were used in all subsequent analyses.

To identify secondary piRNAs produced by the ping-pong amplification cycle, all genomic mapping positions of representative piRNAs were obtained using bowtie2 (v2.4.4, parameters: -a, --score-min L,0,0). For each alignment, the genomic 5’ end and strand were extracted in R using the GenomicAlignments (v1.46.0) and GenomicRanges (v1.62.1) packages [33]. Plus- and minus-strand 5’ ends were compared within each scaffold to identify opposite-strand pairs separated by 0–30 nucleotides. piRNA pairs were deduplicated by sequence identity, and overlap frequencies were obtained for each distance. Enrichment at each overlap distance was assessed using a local z-score computed from a 5-nucleotide window around the focal distance. SeqLogos were generated with the R packages ggseqlogo (v0.2) [34] and ggplot2 (v3.5.1) [35].

Two small RNA-seq libraries from eggs of virgin females (unfertilized eggs from virgin females), dissected before being oviposited, were included to study the maternal transmission of piRNAs (hereafter referred to by the previously used dataset abbreviation NFE [36]; GSE87031) and were processed using the same workflow as the other ovarian libraries.

### piRNA expression analysis in small RNA libraries

Read counts were normalized using the DESeq2 median-of-ratios method (DESeq2 v1.38.3) [37]. The resulting DESeq2-normalized counts were used for downstream quantitative analyses, such as the application of expression thresholds. For visualization analyses, variance-stabilized transformed (VST) values were obtained using the varianceStabilizingTransformation function from DESeq2. VST values were used for Principal Component Analysis (PCA) and for heatmap generation (pheatmap v1.0.12). For the computation of Shannon’s diversity index, representative piRNA raw counts were converted to relative abundances by dividing each piRNA’s count by the total piRNA count of its corresponding library. Shannon diversity was then calculated from these proportional values using the vegan R package (v2.6-4). Differential expression analysis between contiguous ovarian development stages was performed with DESeq2 (FDR < 0.05; log_2_FC > 2 | < −2) [37].

### Characterization of piRNA genomic origins

The genomic origins of piRNAs were assessed by mapping the representative piRNA sequences to the *B. germanica* genome using Bowtie2 (v2.4.4, parameters: -a, --score-min L,0,0) [31], reporting all valid alignments for each sequence. Aligned reads with one or more mismatches were filtered out using samtools. The quantification of piRNAs mapped to genomic features (5’UTR, CDS, 3’UTR, introns, and TEs) was performed using the featureCounts function implemented in the R package Rsubread (v2.12.3, parameters: minOverlap = 25, countMultiMappingReads = T, fraction = T) [38]. Additionally, strandSpecific = 1 was applied to count sense mappings and strandSpecific = 2 for antisense mappings. The piRNA counts mapping to each genomic feature were normalized using TPM, accounting for the potential overrepresentation of piRNAs in longer genomic features. Gene Ontology (GO) terms for *B. germanica* genes were obtained using eggNOG-mapper (version emapper-2.1.12) based on protein sequence orthology [39,40].

### Functional validation

The piRNA_27379 levels were reduced using a chemically unmodified antisense oligonucleotide (ASO: 5′-GATTTACCAGACTCTGGGATCCTACGTCG-3′) designed to encompass the entire piRNA sequence. As a control, a Mock sequence was generated by combining the recognition sites of four restriction enzymes (XbaI, HindIII, KpnI, and BamHI) and adding four extra nucleotides to match the ASO length (5′-TCTAGAAAGCTTGGTACCGGATCCCAGGT-3′) [10]. To assess ASO specificity, we scanned both genomic strands for reverse-complement matches to each oligonucleotide (Mock and piRNA_27379). No complementary sequence was found for Mock. For the ASO targeting piRNA_27379, we detected a single complementary region, corresponding to the piRNA_27379 production site. We also searched the genome for sequences complementary to several 15 nt fragments corresponding to parts of the piRNA_27379-ASO or Mock, and found no additional matches beyond the aforementioned piRNA_27379 locus.

Based on previous data [10], newly emerged adult females were treated with 1 μL of a solution containing 15 μg/μL of ASO (hereafter referred to as ASO-treated) or Mock, in water, injected into the ventral part of the abdomen using a Hamilton® 75 N syringe. Ovaries were dissected on day 5. One ovary was processed to observe the phenotype, while the other was kept at −80 °C to later extract the small RNAs. After dissection, the basal ovarian follicle was measured, and ovaries were immediately fixed in paraformaldehyde (4% in PBS) for 2 h. Washing samples were performed as previously described [41]. Ovaries were incubated at room temperature for 20 min in 300 ng/ml phalloidin-TRITC (Sigma), then for 5 min in 1 µg/ml DAPI (Sigma) in PBT, to visualize F-actin and nuclei, respectively. After three washes with PBT, ovaries were mounted in Mowiol (Calbiochem, Madison, WI, USA) and observed using a Zeiss AxioImager Z1 microscope (Apotome) (Carl Zeiss MicroImaging).

Data are expressed as mean ± standard error of the mean (S.E.M.). Statistical analyses were performed using GraphPad Prism version 8.1.0 for Windows (GraphPad Software). Data were assessed for normality using the Shapiro-Wilk test. All datasets passed the normality test; therefore, no data transformation was required. For comparisons between two independent groups, we first conducted an F-test to evaluate the equality of variances. Based on the F-test outcome, statistical significance was determined using either a standard unpaired Student’s t-test (equal variances) or Welch’s t-test (unequal variances). Results with a p-value < 0.05 were considered statistically significant.

## Results

### The piRNAs in the *Blattella germanica* ovary

Since piRNAs are primarily derived from genomic regions associated with TEs, we examined the *B. germanica* genome to determine the frequency of TEs in this species. Our annotation of the total repetitive content of the *B. germanica* genome identified 47.65% of the assembly as repetitive sequences. DNA transposons and LINEs are the most representative TE orders, spanning 10.93% and 9.71% of the genome, respectively (S2 Table). The genome-wide proportions of other TEs, such as LTRs, SINEs, and Rolling Circles, are much lower (0.49%, 0.45%, and 0.12%, respectively). Additionally, we identified other types of repeats, including simple repeats, microsatellites, low-complexity repeats, and unclassified repeats, which together account for 25.95% of the genome.

We obtained 22 small RNA libraries from nymphal and adult *B. germanica* ovaries at different developmental stages, generating an average of 13.13 ± 2.36 million reads per library. We selected reads ranging from 26 to 31 nucleotides, obtaining an average of 3.54 ± 0.80 million reads per library, of which 65.31% mapped to the reference genome (S3 Table). After collapsing 3’ variants, we obtained 3,931,263 unique sequences. Given the large number of lowly expressed sequences, we performed a filtering step to remove sequences with fewer than 10 identical reads across samples. This yielded a final set of 300,091 unique sequences, representing the *B. germanica* ovarian piRNA repertoire.

Most of the *B. germanica* piRNAs (78.76%) are 28–29 nucleotides long, with 28-nucleotide piRNAs being the most common length (45.34%; Fig 2A), a distribution consistent across all libraries and replicates (S1 Fig). A smaller proportion of putative piRNAs are 27 nucleotides long (12.85%), while the rest correspond to sequences of 26, 30, and 31 nucleotides (Fig 2A). As expected, the piRNA sequences display the typical U-bias at the first 5’ nucleotide (Fig 2B). The search for piRNAs participating in the ping-pong amplification pathway revealed a strong enrichment in 5’-5’ 10-nucleotide overlaps between piRNAs mapping to opposite strands in all ovarian stages (Fig 2D). We detected 11,078 piRNAs exhibiting the ping-pong signature, mostly with a length of 28–29 nucleotides (Fig 2C), and showing the typical A-bias at the 10th nucleotide (Fig 2E).

**Fig 2:**
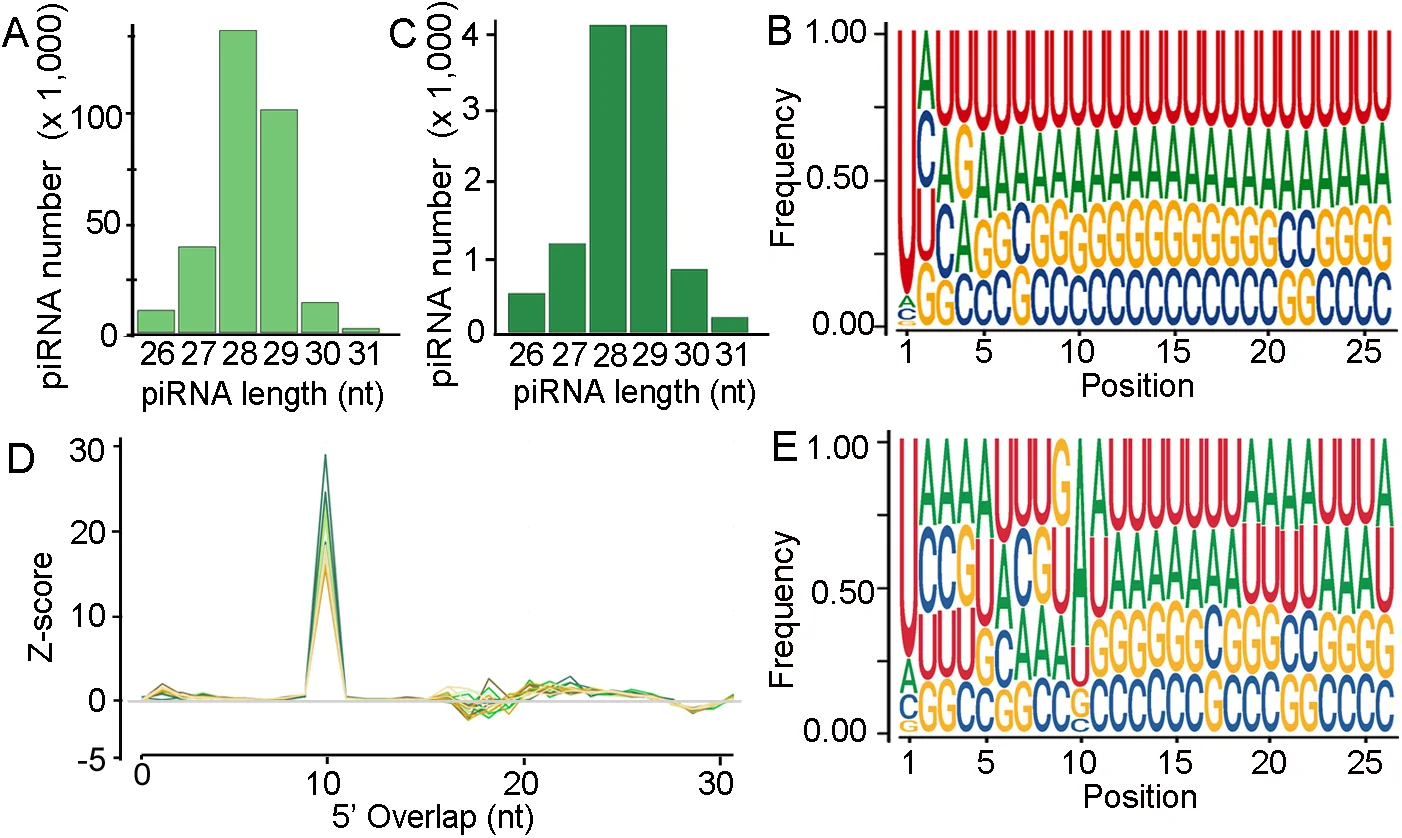
piRNAs identified in the *Blattella germanica* ovary. **A.** Number of *B. germanica* piRNAs distributed by length (nt: nucleotides). **B.** Sequence logo for all identified piRNAs showing the nucleotide frequency in each position, with a clear bias for an uracil (U) in the first 5’ position. **C.** Number of *B. germanica* secondary pathway piRNAs by length (nt: nucleotides). **D.** Line plot of z-score enrichment across 5’ overlap lengths between piRNAs. For each library, z-scores were calculated at every overlap distance. All libraries show a sharp peak at 10 nt, characteristic of the ping-pong amplification signature. **E.** Sequence logo displaying the nucleotide frequency of piRNAs from the secondary pathway, showing the uracil (U) and adenine (A) biases in the 1^st^ and 10^th^ positions, respectively.

### piRNA expression dynamics in the *B. germanica* ovary

The dynamics of piRNA expression in the ovaries during the first gonadotropic cycle*,* were analyzed using PCA (Fig 3A). The analysis revealed a clear divergence of samples along the developmental timeline, reflecting substantial changes during BOF development (Fig 3A). The first principal component (PC1) accounted for 44% of the variance, while the second (PC2) explained 27%. Ovaries of the last nymphal instar and newly emerged adults (AD0) formed a tight cluster, indicating that piRNA expression remains highly similar across those stages. Within this cluster of previtellogenic ovaries, the earliest stage (N6D0) is slightly separated along the first principal component (PC1), suggesting a minor shift in piRNA expression following emergence to the last nymphal instar. At this moment, the BOF are previtellogenic and undergoing capacitation to enter a vitellogenic stage, while the follicular epithelia exhibit a high degree of proliferation (Fig 1A).

**Fig 3:**
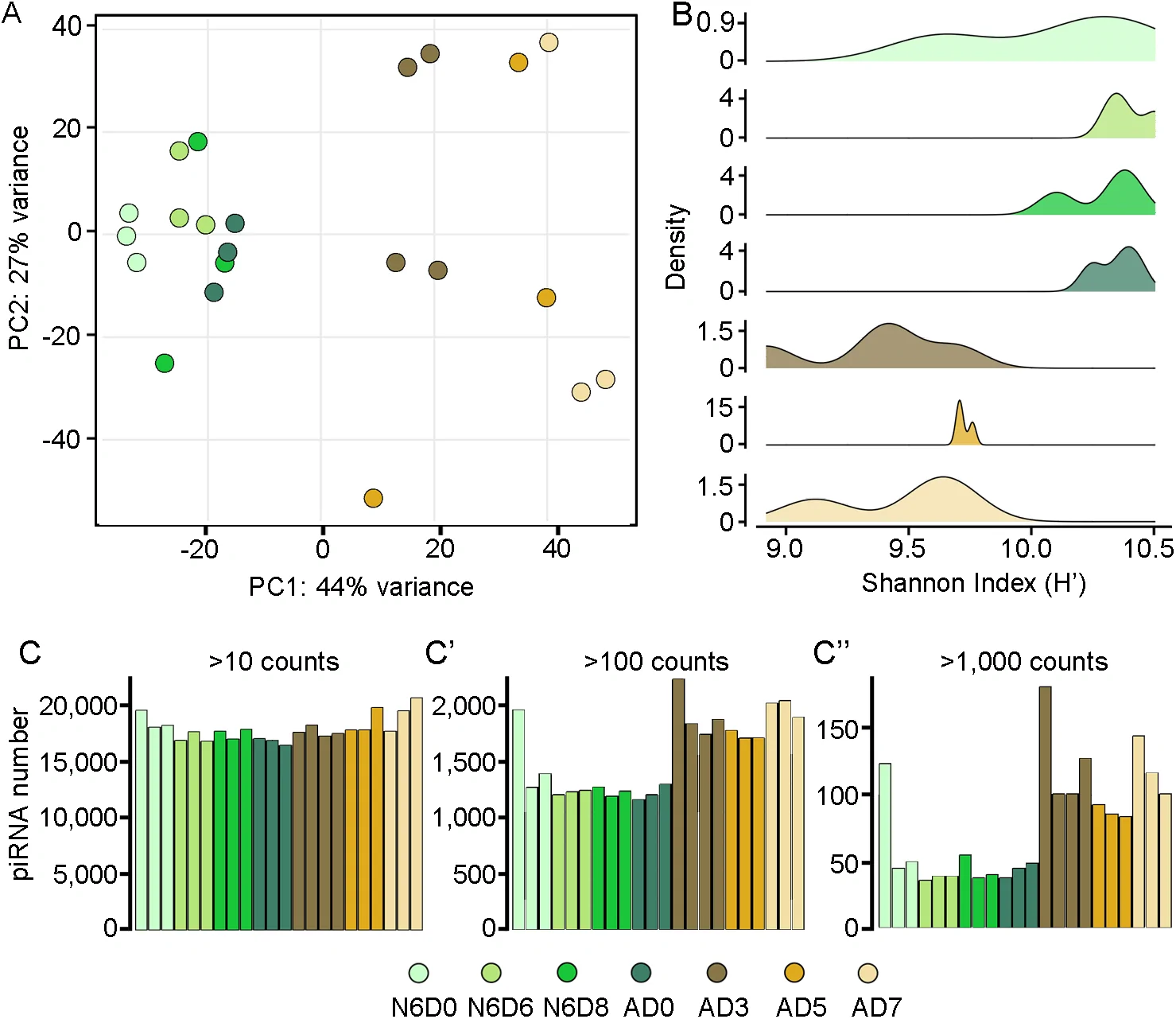
General piRNA expression dynamics in the *Blattella germanica* ovary. **A.** Principal component analysis showing the distribution of ovarian piRNA libraries of last instar nymphs and adults. **B.** Density plot depicting the Shannon’s diversity index (H’) distribution of ovarian piRNA stage libraries. **C.** Number of piRNAs with expression over 10 counts, 100 counts (C’), and 1,000 counts (C”) distributed by stage in each library. N6 indicates last instar nymphs, and A indicates adults. The letter D, followed by a number, indicates the age of the individual in days within the instar.

In contrast, samples of AD3, AD5, and AD7 ovaries showed a broader dispersion along the PC1, indicating substantial changes in piRNA expression over oocyte maturation (Fig 3A). The PCA analysis revealed a clear difference between previtellogenic (N6D0 to AD0; immature) and vitellogenic (AD3 to AD7) ovaries, considering piRNA content and expression. These differences appear to be especially marked between immature and AD3 ovaries, as well as between AD3 and AD5 ovaries (Fig 3A). The dispersion along PC2 evidenced variability across replicates in vitellogenic ovaries. This variability may be attributed to the physiological transitions occurring in the BOF, mainly associated with the changes in the follicular cell program that happen over brief time periods at the end of the gonadotropic cycle (see Fig 1A).

The analysis of piRNA diversity using Shannon’s diversity index (H’) revealed two distinct profiles (Fig 3B), which correspond to the clustering observed in the PCA. Libraries from immature ovaries exhibited greater piRNA diversity, as reflected by higher H’ values (Fig 3B), consistent with a more even distribution of expression across different piRNAs. In contrast, vitellogenic ovaries exhibited reduced piRNA diversity, suggesting that during these stages, a smaller subset of distinct piRNAs is expressed at higher levels (Fig 3B).

We examined piRNA abundances across a range of expression thresholds to clarify how the expression of several piRNAs contributes to the patterns seen in the PCA and Shannon’s diversity analysis (Figs 3A and 3B). The number of piRNAs with expression levels above 10 DESeq2-normalized counts was consistent across all ovarian libraries (Fig 3C). However, when the threshold was raised to 100 DESeq2-normalized counts, a clear distinction emerges between mature and immature ovaries (Fig 3C’). The highest expressed piRNAs are more abundant in mature ovaries. When the threshold was extended to 1,000 DESeq2-normalized counts, the difference between mature and immature ovaries became even more evident, with mature ovaries containing about twice the amount of piRNAs compared to those of immature stages (Fig 3C”).

Differential expression analysis between consecutive stages of ovarian development revealed significant changes in piRNA expression across these stages (Figs 4 and S2). In the first transition, from N6D0 to N6D6, only 76 piRNAs were differentially expressed, with 30 upregulated and 46 downregulated in N6D6 ovaries (see S2 Fig). Most of these piRNAs maintain their expression in immature ovaries. Interestingly, the subsequent transitions (N6D6 to N6D8 and N6D8 to AD0) showed very small changes in piRNA expression, with only 0 and 1 differentially expressed piRNAs, respectively. All of these suggest the presence of a stable set of piRNAs in immature ovaries, likely essential for maintaining cellular functions, particularly in the BOF.

**Fig 4:**
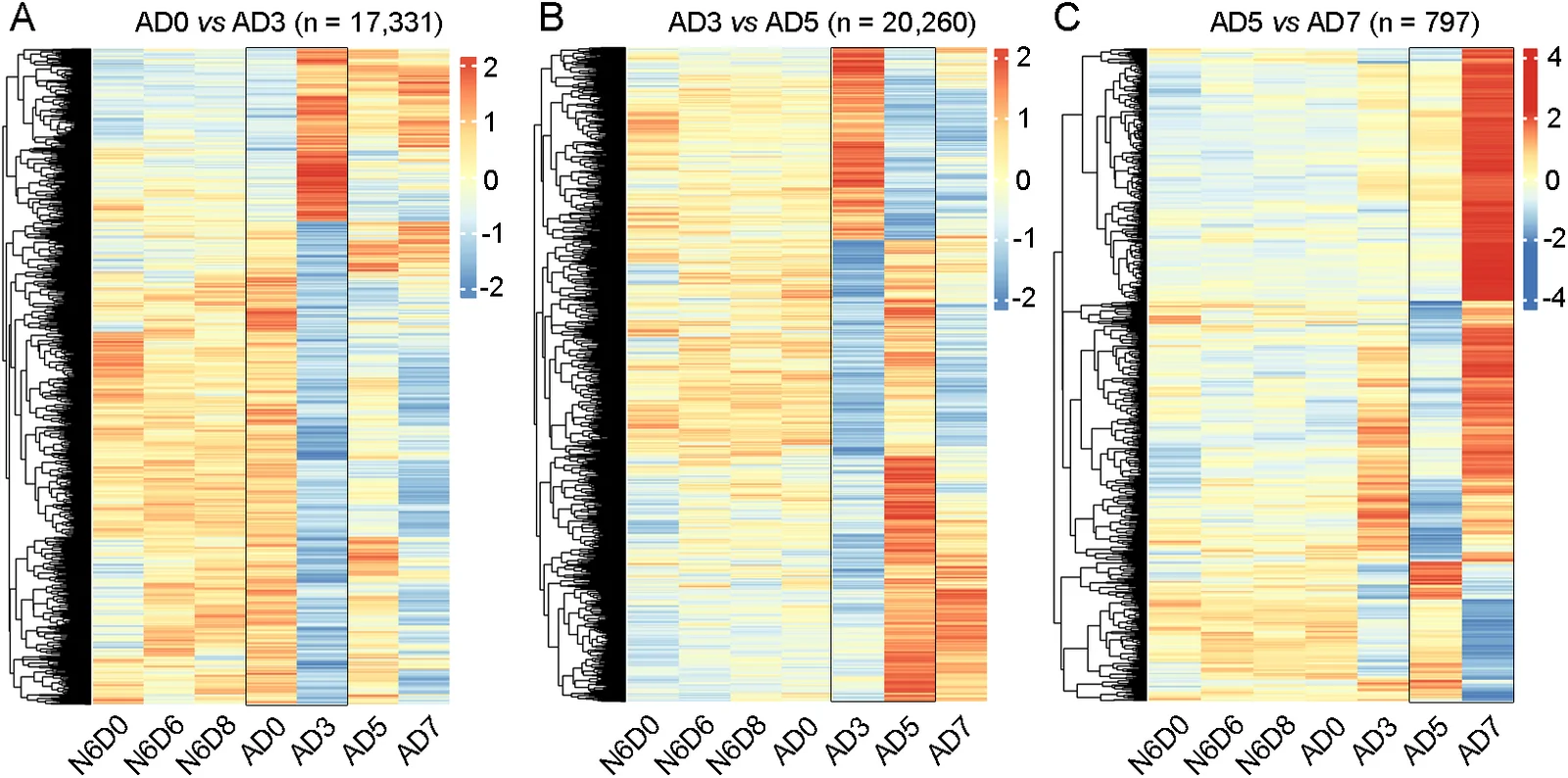
Differential expression of *Blattella germanica* piRNAs throughout ovary maturation. Heatmap Plots showing the piRNAs differentially expressed in the transitions: 0-day-old adult (AD0) to AD3 **(A)**, AD3 to AD5 **(B)**, and AD5 to AD7 **(C)**; with a log_2_FC > 2 | < −2 and a False Discovery Rate (FDR) < 0.05. Expression of the differentially expressed piRNAs is depicted for all stages (N6D0 to AD7) to show their expression profile throughout the whole ovary maturation process. In the transition N6D0 to N6D6, only 76 piRNAs were found to be differentially expressed (see S2 Fig), whereas the transitions N6D6 to N6D8 and N6D8 to AD0 resulted in 0 and 1 DE piRNAs, respectively.

The transition from an immature to a mature BOF (AD0 to AD3) is critical in the *B. germanica* ovary. In AD3, the follicular cell proliferation in the BOFs begins to arrest, while oocytes grow by accumulating storage proteins, mainly vitellogenin (Fig 1A). During this transition, a significant number of piRNAs (17,331) are differentially expressed (Fig 4A). One major group is downregulated in AD3 ovaries, and some of these piRNAs increase in expression again as the BOFs continue maturing. In contrast, a second group of piRNAs expressed at lower levels in previtellogenic ovaries shows elevated expression in AD3 and maintains these levels until the end of the gonadotropic cycle.

During the transition from AD3 to AD5, JH levels in the hemolymph increase, and 20E begins to accumulate in the ovary (Fig 1A). The follicular cells in the BOF arrest cytokinesis and become binucleated, entering polyploidy (Fig 1A). At this moment, a notable change in piRNA expression occurs, with 20,260 piRNAs identified as differentially expressed (Fig 4B). Many of the piRNAs appear upregulated in AD5 and mostly maintain their expression levels in AD7, suggesting a specific role in the later stages of the gonadotropic cycle. Conversely, a group of piRNAs is downregulated in AD5. These downregulated piRNAs maintain their low expression levels until oviposition.

The final transition, from AD5 to AD7, is critical for completing the gonadotropic cycle. In the ovaries of 7-day-old females, follicle cells in the BOF undergo endoreplication in specific genomic regions, leading to upregulation of chorion genes (Fig 1A; [21]. During this transition (Fig 4C), a total of 797 piRNAs are differentially expressed, exhibiting a wide range of fold changes. Notably, 78.50% of these piRNAs are upregulated, with an average fold change of 3.3, ranging from 2.0 to 22.6 (Fig 4C), reaching the highest expression levels recorded across all stages. In contrast, at AD7, only a limited number of piRNAs are downregulated; 171 piRNAs show a decrease in expression when compared to AD5, with fold changes ranging from 2.0 to 6.7 (Fig 4C).

The significant increase in upregulated piRNAs in AD7 (Fig 4C) raises an important question regarding their function: Are these piRNAs acting within the ovary, or are they being transferred to the egg? To address this question, we utilized small RNA libraries corresponding to eggs from virgin females (NFE: non-fecundated eggs [14,37]. We analyzed whether the piRNAs present in the NFE samples were also present in other ovarian stages.

Our analysis revealed that there are 201,927 piRNAs present in mature ovaries that are not transferred to the egg. Additionally, we identified a small subset of 10,749 piRNAs that were not detected in any of the ovarian libraries with mature BOF (S3A Fig). This subset represents approximately 3.49% of the total piRNAs present in the NFE samples and is generally found at low read counts (S3B Fig), which likely contributed to their undetectability in other libraries. In contrast, the 94,748 piRNAs identified in the NFE samples that are also present in mature ovaries appear in greater proportions (S3C Fig). This indicates that a large number of ovarian piRNAs are likely involved in regulating oocyte maturation and maintaining the appropriate developmental program during this process.

### The genomic origins of piRNAs

To better understand the functions of piRNAs in *B. germanica*, we investigated the genomic regions where piRNAs were mapped without any mismatches. To ensure valid comparisons between different features and samples, the number of mapped locations was normalized using TPM, accounting for the potential overrepresentation of piRNAs in longer genomic features. The adjusted values were then used to calculate the mapping proportion of piRNAs to each feature within each sample.

As expected, the main sources of piRNAs correspond to TEs (Fig 5A), with 88.90% of the ovarian piRNAs mapping to TE sequences. We analyzed which TEs piRNAs map to and whether they align to the TE sense or antisense strand (Figs 5A, 5C, and S4). Among Class I TEs, piRNAs are in a relatively stable density throughout all stages of ovarian development (Figs 5A, 5C and S4), except for those piRNAs mapping SINEs in the sense strand, which are less abundant in ovaries at the end of the gonadotropic cycle, becoming significantly reduced in ovaries from AD7, just before oviposition. Within this Class I, we found that the largest number of piRNAs mapped to LINEs, in both sense and antisense strands (Figs 5A, 5C and S4). Conversely, among Class II TEs, the proportion of piRNAs mapping to DNA TEs increases significantly at the end of the cycle, compared to the rest of the days (S4 Fig), whether they map to the sense or the antisense strand. The piRNAs mapping to the antisense strand of RC decrease significantly in the last days of the gonadotropic cycle (Figs 5C and S4).

**Fig 5:**
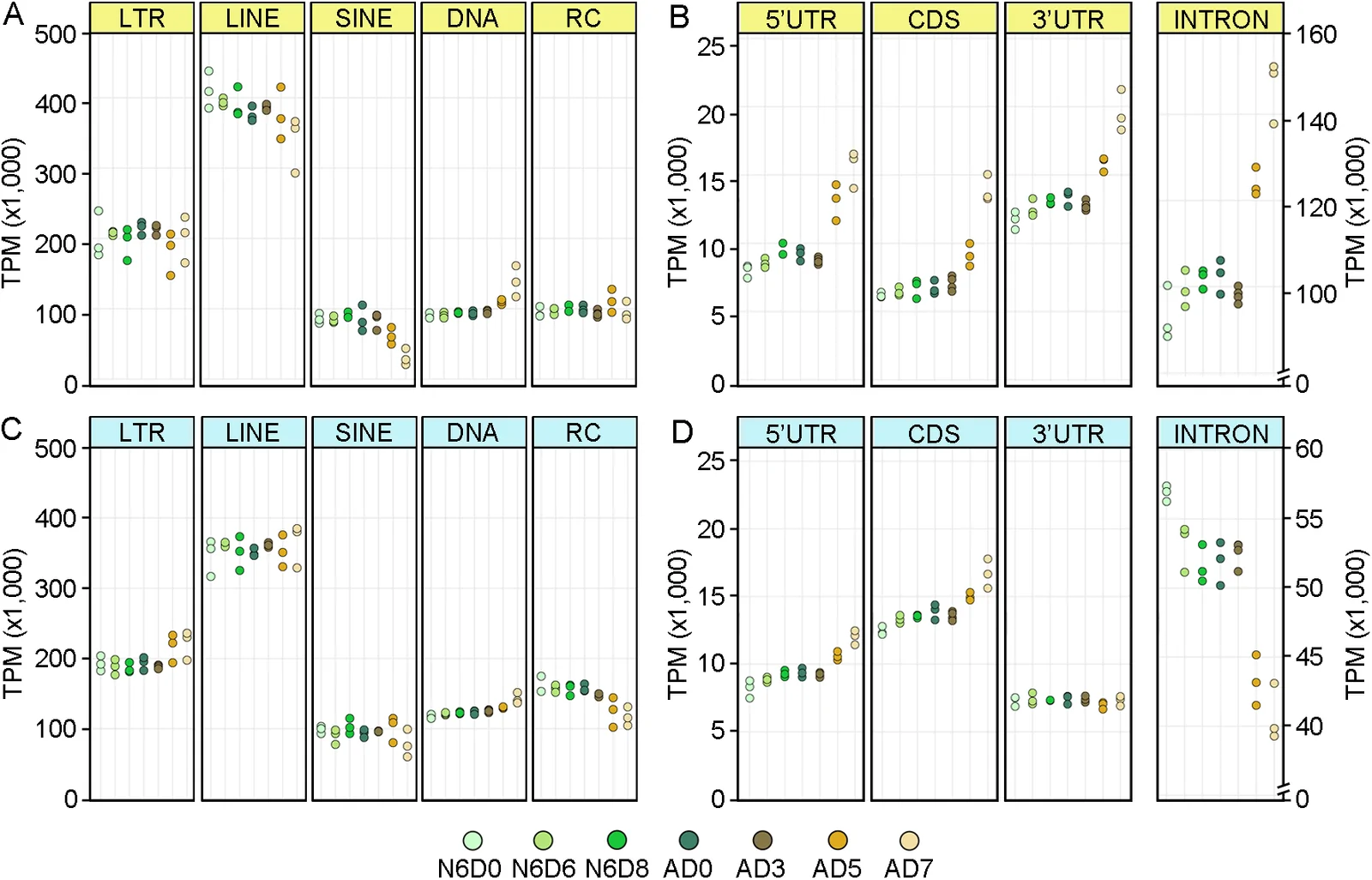
Genomic mapping sites of ovarian piRNAs in *Blattella germanica.* **A.** TPM of piRNAs mapping to the five main TE orders (LTR, LINE, SINE, DNA, and RC) in the sense strand. **B.** TPM of piRNAs mapping to the genic regions (5’UTR, CDS, 3’UTR, and Introns) in the sense strand. **C.** TPM of piRNAs mapping to the five main TE orders (LTR, LINE, SINE, DNA, and RC) in the antisense strand. **D.** TPM of piRNAs mapping to the genic regions (5’UTR, CDS, 3’UTR, and Introns) in the antisense strand. N6 indicates last instar nymphs, and A the adults. The letter D, followed by a number, indicates the age of the individual in days within the instar. In yellow, piRNA mappings in the sense strand. In blue, piRNA mappings in the antisense strand of the corresponding feature.

In addition to TEs, a significant portion (11.11%) of piRNAs map to genic regions (3.20% in UTRs and CDS, 7.90% in introns), in both strands (Figs 5B, 5D and S5). Showing significant changes across the different stages as the BOF matures in the ovary (Fig 5B, 5D, and S5). In the later stages of the gonadotropic cycle, in AD5 and AD7, there is a significant increase in piRNAs mapping to CDS and 5’UTRs (S5 Fig) in both strands. These changes are also occurring in those piRNAs mapping to the sense strand of 3’UTRs, suggesting an increase in regulatory functions for piRNAs in the ovaries at these ages. The high number of piRNAs mapping to introns is stable in immature ovaries. However, in ovaries from AD5 and AD7, a significant increase in piRNAs in the sense strand occurs, while in the antisense strand, with a much lower number, a clear decrease in piRNAs is observed (Figs 5B, 5D and S5).

Additionally, when analyzing the piRNAs mapping to genic regions such as CDS, 5’UTR, and 3’UTRs, we identified a small group of piRNA pairs that align to opposite strands in the same genomic locus with a 10-nucleotide difference in their start positions, similar to those described by Jensen and collaborators [42]. The analysis found a total of 18 genes presenting intrinsic overlapping pairs. The roles of these genes cover various biological processes, including autophagy and lysosome positioning (3/18), chromatin regulation (3/18), ribosome biogenesis (1/18), transcription regulation (1/18), and transposon-derived proteins (4/18). In comparison, six genes encode proteins with unknown functions.

### piRNA function in *Blattella germanica* ovary

The significant changes observed in the expression of piRNAs in mature ovaries led us to validate some of them using qRT-PCR. We selected four piRNAs with distinctly different profiles in the libraries (see Fig 6, orange histograms) and quantified their expression by qRT-PCR across all days of the first gonadotrophic cycle (see Fig 6, green histograms). As shown in Fig 6, the qRT-PCR expression profiles closely align with those identified in the analyzed libraries (see Fig 6, brown histograms).

**Fig 6:**
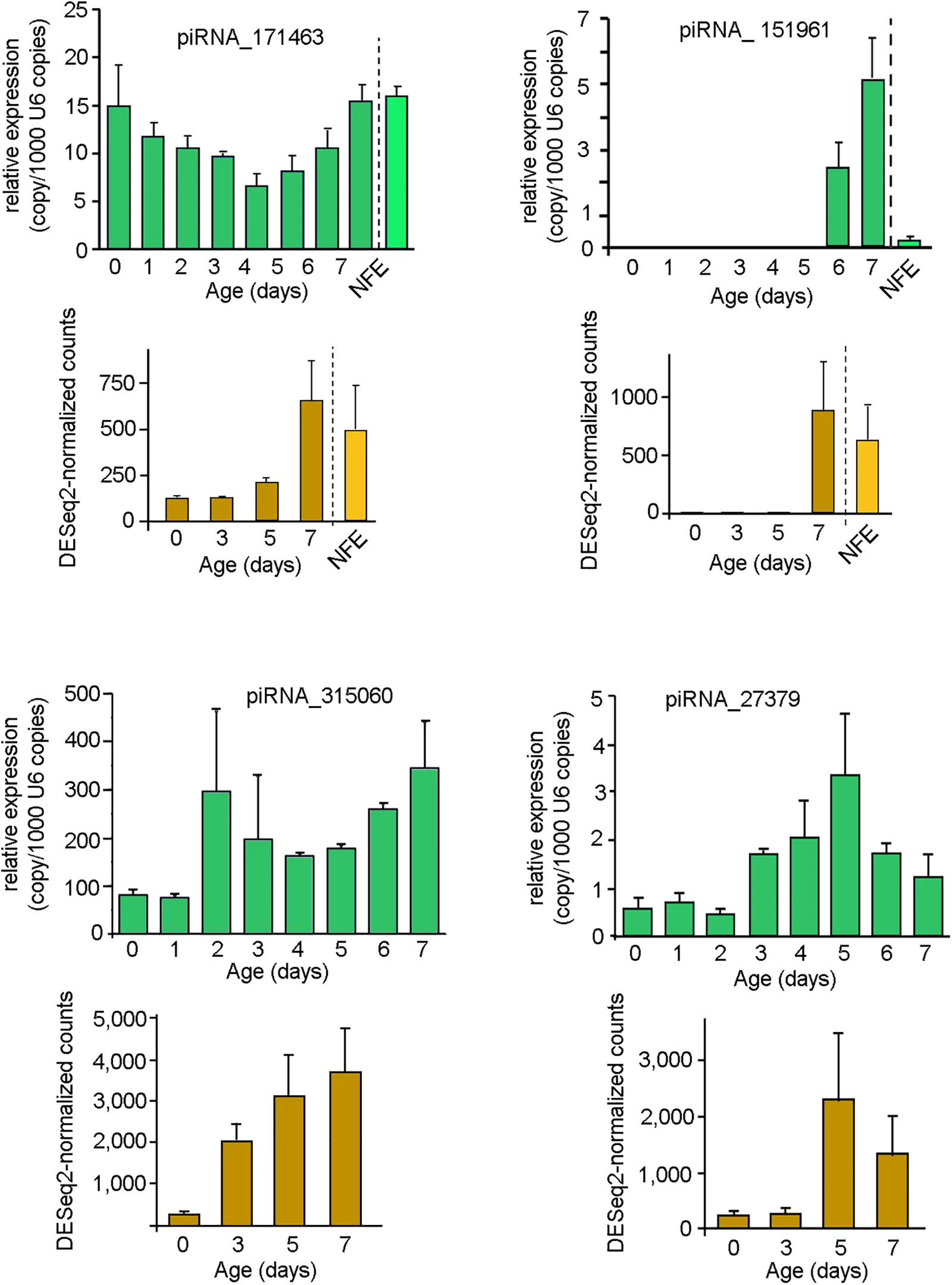
piRNA expression in *Blattella germanica* ovaries. Library validation. RT-qPCR expression profile of four piRNAs highly expressed in adult ovaries during the first gonadotropic cycle (green). Expression in non-fecunded eggs (NFE) is included for two piRNAs. Data represent copies of piRNA per 1000 copies of *U6*, and are expressed as the mean ± S.E.M. (n = 3). Data from the ovarian small RNA libraries corresponding to mature ovaries and NFE [37] are included (orange). Each value represents the average of normalized counts, calculated using the median-of-ratios method from DESeq2. Data are shown as mean ± S.E.M. (n = 3, except Ad3 n = 4). The vertical dotted line separates the expression in NFE from the expression in ovaries.

Among them, we selected piRNA_27379 to study its function in *B. germanica* ovaries. Newly emerged adult females (AD0) were treated with an ASO against piRNA_27379 or with a Mock oligonucleotide, and ovaries were dissected five days later. In ASO-treated females, piRNA_27379 expression levels were significantly reduced (Student-test, p = 0.0106), compared with Mock-treated females (Fig 7A). BOF size in ASO-treated females was significantly smaller (0.532 ± 0.007 mm) than in Mock females (1.578 ± 0.030 mm; Fig 7B and D; Welch’s t-test, p < 0.0001), evidencing a delay in BOF growth.

**Fig 7:**
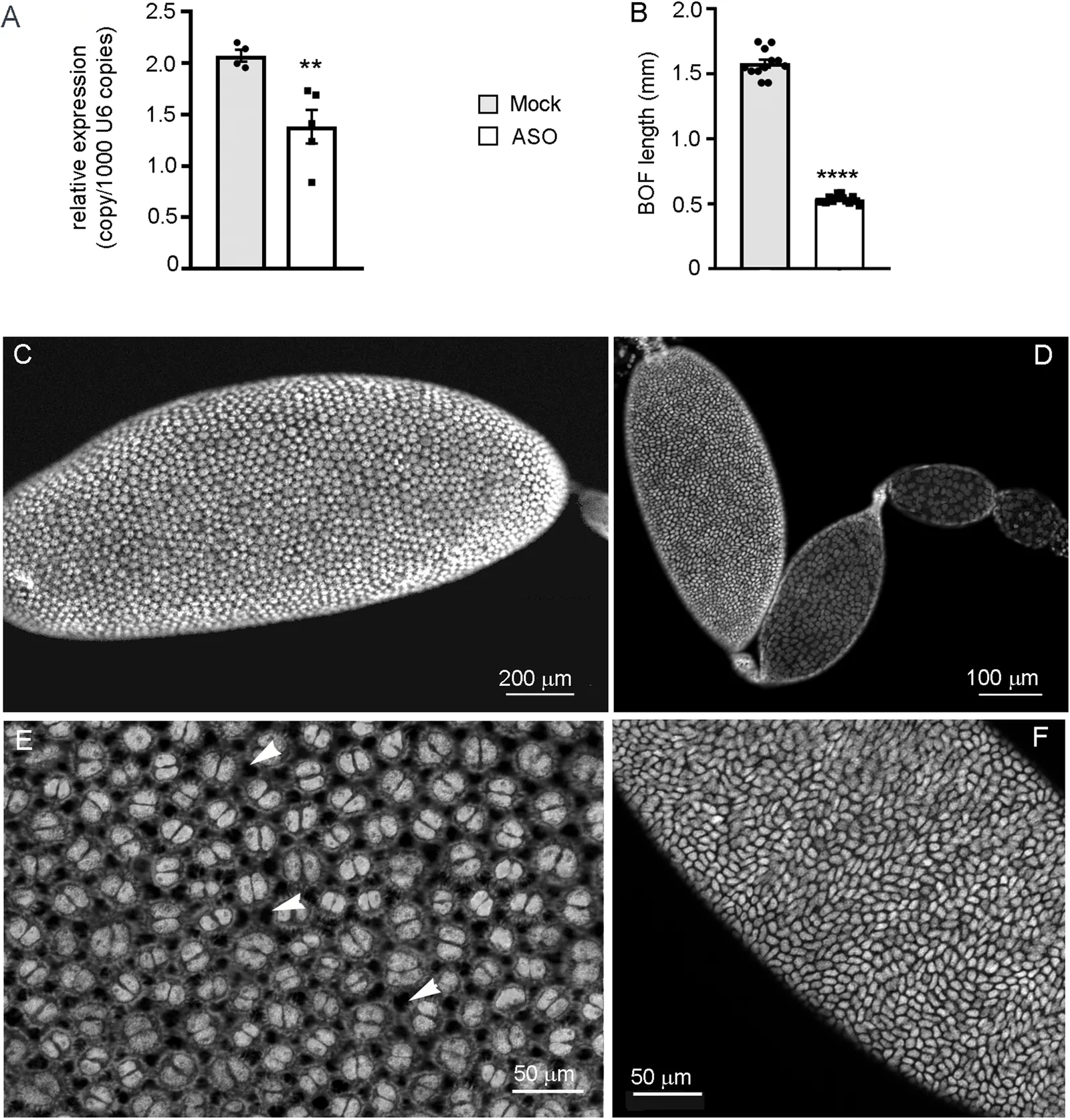
Effects of piRNA_27379 depletion in the ovaries of *Blattella germanica.* **A.** Expression levels of piRNA_27379 in *B. germanica* adult ovaries. Females were injected with Mock (n = 4) or ASO (n = 5) on the day of adult emergence, and ovaries were dissected 5 days later. Data represent copies of the piRNA per 1000 copies of *U6* and are expressed as the mean ± S.E.M. The asterisks indicate statistically significant differences (p = 0.0106). **B.** Basal ovarian follicle length (BOF) in 5-day-old females treated with Mock and ASO (3 BOF per female were measured). Data are expressed as the mean ± S.E.M. The asterisks indicate statistically significant differences (p < 0.0001). **C**. Basal ovarian follicle (BOF) from a Mock-treated female. **D**. Ovariole from an ASO-treated female, which is smaller than in Mock females, indicating a delay in BOF growth. **E.** Follicular epithelium from a 5-day-old Mock-treated adult female, showing the follicular cell distribution. Cells are binucleated, with big nuclei, distributed uniformly, and the intercellular spaces are open (arrowheads). **F.** Detail of the BOF in an ovariole from an ASO-treated female. The image is at the same magnification as E. The nuclei in the follicular cells are smaller than in controls, and there are no intercellular spaces between the cells, indicating a delay in the follicular cell program. F-actin microfilaments were stained with phalloidin-TRITC, and DNA was stained with DAPI.

In 5-day-old Mock-treated adult females, BOF binucleated follicular cells exhibit uniform distribution, with nuclei of the same size and shape (Fig 7C and E), and with the intercellular spaces opened (Fig 7E, arrowheads) to allow the storage proteins from the hemolymph to reach the oocyte membrane. Conversely, in ASO-treated females, the follicular cells in the BOF were small and mononucleated, with small nuclei (Fig 7F). The cells were very tightly packed, leaving no spaces between them. All of that indicates a delay in oocyte growth and maturation, which affects the follicular cell program.

Taken together, our data demonstrate that piRNA expression undergoes coordinated changes in ovaries during the transition from previtellogenic to vitellogenic stages, suggesting that these piRNAs may play essential roles in the gonadotropic cycle to complete BOF development.

## Discussion

piRNAs are a class of molecules with gene regulatory functions targeting TEs and RNAs (including mRNAs and long non-coding RNAs) [6]. In arthropods, piRNA expression has been reported in a variety of gonadal and somatic tissues [6,43,44]. Among these, the ovaries, with both somatic and germline cell lineages, exhibit the highest abundance of piRNAs [45–47].

TEs constitute the main genomic source of piRNAs. Petersen et al. [48] and Ylla et al. [49] suggest that TE content tends to increase with genome size in insects, although considerable variability among species has been documented. For example, in the wasp *Nasonia oneida*, 32.29% of the genome consists of repetitive sequences [50], whereas in the honey bee *Apis mellifera*, only 9.46% is repetitive, with TEs accounting for just 3% [51]. In Diptera, TE content also varies widely, ranging from 20% in *D. melanogaster* [52] to 47.72% in *Aedes aegypti* [53]. A similar range is observed in hemimetabolous insects. Within Orthoptera, TE content differs markedly: TEs represent 28.94% of the genome in the cricket *Gryllus bimaculatus* [49] and 65% in the locust *Locusta migratoria* [48]. In the cockroach *B. germanica*, our analysis indicates that 47.65% of the genome is composed of repetitive sequences, of which 21.70% correspond to annotated TEs. Among these, DNA transposons are the most abundant, accounting for 10.93% of the genome. This pattern contrasts with that observed in Gryllidae, where LINE elements predominate, constituting 20.21% of annotated TEs in *Laupala kohalensis* [49]. In other species, such as *D. melanogaster* and *Tribolium castaneum*, LTR retrotransposons are the most abundant TE class [54,55]. Notably, although SINEs are present across insect orders, they generally remain scarce [48].

Given the abundance of TEs in insect genomes, one would expect a similarly high number of piRNAs. This is the case in species such as *Rhodnius prolixus* [56] and *Aedes albopictus* [57], in which approximately 36 million and 12 million piRNAs have been reported, respectively. These observations suggest that more stringent criteria should be applied to accurately identify biologically active piRNAs and enable studies of their potential regulatory roles [58]. By applying stringent filtering conditions in *B. germanica,* collapsing identical sequences with different trimming levels that map to the same genomic locus, and selecting the most highly expressed sequence, we substantially reduced the number of previously identified piRNAs [14]. The resulting dataset of 300,091 piRNAs enabled us to analyze their dynamic expression in ovaries, and the PCA provides initial evidence for a role of piRNAs in ovarian maturation in *B. germanica*, as it clearly separates previtellogenic from vitellogenic ovaries. This separation appears to be driven by differences in both the origin and abundance of piRNAs at each stage.

Although the number of piRNAs associated with certain transposable element (TE) families remains relatively constant throughout the gonadotropic cycle, substantial variations occur during the last days. For instance, piRNAs mapping to the sense strand of SINEs and the antisense strand of retrotransposons (RC) show a notable decrease, while piRNAs mapping to DNA transposons—regardless of strand orientation—exhibit a marked increase on the last day of the cycle. These findings suggest that TEs play an active role at the end of the cycle, either serving as a source of piRNAs or acting as a target for depleting piRNAs.

In *B. germanica*, approximately 3.5% of piRNAs map to genic regions (CDS and UTRs), which is relatively low compared with *L. migratoria*, another species with panoistic ovaries, where 11% of piRNAs have a genic origin, including 8% mapping to CDS regions [44]. In species with meroistic ovaries, such as the bug *Oncopeltus fasciatus* and the bumblebee *Bombus terrestris*, the proportion of piRNAs mapping to CDS is notably higher (25% and 48%, respectively), whereas the proportion mapping to UTRs remains relatively constant [44].

In *B. germanica*, we observed significant stage-dependent changes in the abundance of piRNAs mapping to genic regions. Notably, at the end of the gonadotropic cycle (in 5- and 7-day-old adult females), the greatest variability was detected in piRNAs mapping to the 5’ UTR, CDS, and introns, on both the sense and the antisense strand. In contrast, for piRNAs mapping to the 3’UTR, the differences at the end of the cycle were restricted to the piRNAs mapping the sense strand, while the piRNAs mapping the antisense strand remain stable.

Analysis of piRNA expression across the different stages of *B. germanica* ovarian development revealed only a small number of differentially expressed piRNAs (76 piRNAs) in immature ovaries (N6D0-AD0), indicating an overall stable piRNA expression at these stages. However, some piRNAs showed significant downregulation immediately after the molt to the final nymphal instar. These changes may be associated with the beginning of ovarian maturation, which begins early in this instar and is characterized by stabilization of follicle number within the ovarioles and the initiation of BOF growth [3,17]. Accordingly, both the composition and expression levels of piRNAs clearly distinguish previtellogenic ovaries (with immature BOFs), including those of newly molted adults, from mature ovaries (Fig 3A). In mature ovaries, the changes in piRNA expression align with the major significant transitions in the follicular epithelium of the BOF. At AD5, a subset of piRNAs is downregulated, suggesting that their functions could be related to the changes in follicular cells program, which become binucleate and polyploid at this stage [3,16]. In AD7, the last day of the gonadotropic cycle, a small set of piRNAs is differentially expressed, with some exhibiting high expression levels.

The functions of piRNAs in various organisms, including humans, have been validated using different types of ASOs. In insects, the use of ASOs to deplete piRNAs is expanding [59], proving useful for regulating piRNA levels and studying their functions [10,60]. Our results, which utilized an ASO to deplete piRNA_27379, align with previous findings [10] where we demonstrated that an ASO against piRNA_3050221 indirectly inhibits the expression of shade mRNA during embryo development. Nevertheless, while ASO-mediated knockdown provides valuable evidence for the biological relevance of these piRNAs, further experiments are needed to confirm whether the observed phenotypes are specifically mediated through canonical PIWI-piRNA interactions.

Taken together, these findings suggest that some insect piRNAs play a role in BOF maturation, being essential in regulating insect oogenesis. Our study unveils a dynamic ovarian piRNA landscape in *B. germanica*, characterized by stable periods and sharp transitions that likely correlate with major endocrine and cellular events of the gonadotropic cycle. Furthermore, we set up the basis for future research into how small RNAs integrate hormonal signalling, follicular cell dynamics, and gene regulation in the ovary of hemimetabolous insects.

## Supporting information

S1 Fig Read length distribution of *B. germanica* small RNA ovarian libraries. For each library, the number of reads with lengths between 18–35 nt after adapter removal are shown. All libraries display a main peak at 28–29 nt, consistent with piRNA length, and a second peak at 22 nt, corresponding to the length of miRNAs.
(JPG)

S2 Fig Differential expression analysis of piRNAs in ovaries of *Blattella germanica* in the transition from N6D0 to N6D6. The heatmap plot depicts the expression profile of differentially expressed piRNAs between N6D0 and N6D6 throughout ovarian maturation, with a log2FC > 2 | < −2 and a False Discovery Rate (FDR) < 0.05.
(JPG)

S3 Fig Maternal transmission of piRNAs in *Blattella germanica.* A. Comparison of piRNA content in the mature ovarian stages (AD3, AD5, and AD7) and unfertilized egg (NFE). The UpSet plot was generated with the R package ComplexHeatmap (v2.26.0) using the “distinct” mode for combination size calculation. B. Number of NFE-exclusive piRNAs at different count levels. C. Number of piRNAs shared between mature ovarian libraries and NFE libraries at different count levels. The count levels shown in B and C were calculated as the log10 of the DESeq2-normalized mean counts in NFE (n = 2).
(JPG)

S4 Fig Mapping sites of *Blattella germanica* ovarian piRNAs in annotated TEs. piRNAs mapping to the different TE orders: LTR, LINE, SINE, DNA, RC, in sense and antisense strands, expressed in TPM. Data is presented as mean ± S.E.M. (n = 3, except AD3 n = 4). The asterisks indicate statistically significant changes: * = p < 0.03, ** = p < 0.01, *** = p < 0.001, **** = p < 0.0001, ns = no significance.
(JPG)

S5 Fig Mapping sites of *Blattella germanica* ovarian piRNAs in genic features. piRNAs mapping genic regions: 5’UTR, CDS, 3’UTR and introns, in sense and antisense strands, expressed in TPM. Data is presented as mean ± S.E.M. (n = 3, except AD3 n = 4). The asterisks indicate statistically significant changes: * = p < 0.03, ** = p < 0.01, *** = p < 0.001, **** = p < 0.0001, ns = no significance.
(JPG)

S1 Table Primers used in the qRT-PCR. F: forward primer, R: reverse primer.
(DOCX)

S2 Table Genome coverage (%) of principal classes of repeated sequences in the B. germanica genome. The total percentage of genome coverage by repeated sequences is 47.64%.
(DOCX)

S3 Table Summary of the Blattella germanica small RNA-seq libraries, available at GEO under accession GSE255380. (XLSX)
