# Supplementary material for: The dynamics of piRNA expression in Blattella germanica ovaries
Source: PLoS One. 2026 Oct 6;21(10):e0347365. doi: 10.1371/journal.pone.0347365 (PMC13641354; doi:10.1371/journal.pone.0347365)
Supplement: Supplementary file 1 [file pone.0347365.s006.docx]

**Table S1.** Primers used in the qRT-PCR. F: forward primer, R: reverse primer.

| **Name** |  | **Primer Sequence (5’ --> 3’)** |
| --- | --- | --- |
| piRNA_171463 | F | TCGAGTTTCACGGAGAGTCCCTCTGAAG |
| piRNA_151961 | F | TCCACGACGATAATATTCTCTGTAATGTC |
| piRNA_315060 | F | TTCTTGAGGGACTTCTGTAGGATCGTAC |
| piRNA_27379 | F | CGACGTAGGATCCCAGAGTCTGGTAAATC |
| Universal (Agilent) | R | GACGAGCTGCCTCAGTCGCATA |
| U6 | F | CGATACAGAGAAGATTAGCATGG |
|  | R | GTGGAACGCTTCACGATTTT |
