# Supplementary material for: The dynamics of piRNA expression in Blattella germanica ovaries
Source: PLoS One. 2026 Oct 6;21(10):e0347365. doi: 10.1371/journal.pone.0347365 (PMC13641354; doi:10.1371/journal.pone.0347365)
Supplement: Supplementary file 2 [file pone.0347365.s007.docx]

**Table S2.** Genome coverage (%) of principal classes of repeated sequences in the *B. germanica* genome. The total percentage of genome coverage by repeated sequences is 47.64%.

| **TE Classification** | **Genome Coverage (%)** |
| --- | --- |
| LTR | 0.49 |
| LINE | 9.71 |
| SINE | 0.45 |
| DNA | 10.93 |
| Rolling Circle (RC) | 0.12 |
| Other (Simple Repeats, Microsatellites, RNA) | 0.25 |
| Unclassified | 25.70 |
